# Edinburgh Surgical Hospital

**Published:** 1830-05

**Authors:** 


### EDINBURGH SURGICAL HOSPITAL.

### *Cases of Excision of the Elbow-joint.* From the *Quarterly Report, by* James Syme, Esq.*

Little more than a year ago, in November 1828, I cut out a carious elbow, for the first time the operation was ever performed in Great Britain. Since then I have operated in six cases. The profession, I am happy to say, seem to be satisfied with the weight of this evidence, and at least two operations of the kind have been lately performed by different practitioners in Edinburgh. Five of my cases have already been put upon record through the medium of this Journal, and I will now relate the other two.

Elizabeth Johnston, æt. fifteen, from Falkirk, entered the hospital on the 26th of August, on account of a disease of the right elbow-joint, which had existed for six months, commenced spontaneously, and increased progressively, notwithstanding the efforts of her medical attendants. It now presented a most formidable appearance, the joint being so much swelled as to measure thirteen inches in circumference, and the arm above being reduced to little more than skin and bone, which made the enlargement seem even greater than it really was. The skin over the olecranon was extensively ulcerated; and at different places, both on the front and back parts of the joint, the probe could be passed into sinuses which extended to the bones. The limb was straight, and nearly immoveable. The discharge was profuse, the pain unceasing, and the irritation so great that the patient's health seemed rapidly sinking. It was plainly necessary to do something effectual for

* Edinburgh Med. and Surg. Journal, April 1830.

her relief, and both Dr. Ballingall and I, though entertaining the most favorable opinion of excision, from what we had seen of its good effects, resolved that any operation short of amputation would be inexpedient in this case, where there was such extensive disease not only of the bones, but also of the soft parts. Being, however, very averse in general to amputating the arm for caries, and feeling particular reluctance to mutilate this unfortunate girl, who was distinguished by the most amiable disposition and interesting appearance, I delayed the operation. In the course of ten days, whether it was owing to a real improvement proceeding from the free vent which had been afforded to the matter by incisions, or was merely the effect of familiarity with the appearance of the joint, I fancied that it was not so hopeless as at first believed, and resolved to make an attempt at excision.

The operation was performed in the manner formerly described, and was attended with very little difficulty, owing to the separation of the surrounding soft parts from the articulating bones, which had been caused by collections of matter. The olecranon was greatly expanded, and, if I may use the expression, completely rotten, so that it crumbled into fragments, which were extracted piecemeal. The radius adhered to the humerus, and was extracted along with it. Before dressing the wound, I observed that the ulnar nerve was partially divided by an oblique incision, and therefore cut it completely across, to avoid the danger of such a wound; and its extremities being then placed in contact, the integuments were stitched together. The patient did extremely well; the wound healed most kindly; the swelling of the joint subsided; she gradually regained its use; and is now, I am happy to understand, restored to perfect health.* For some time after the operation, she complained of coldness and numbness in the ulnar side of the hand, but in process of time got rid of these unpleasant symptoms, probably in consequence of reunion between the extremities of the nerve.

James Page, ætatis eight, was recommended to the Surgical Hospital by Mr. Ferguson, of Auchtermuchty, as a proper subject for excision of the elbow-joint, and was admitted on the 2d January. The right elbow was much enlarged, discoloured, and stiff. There were two sinuses opening on each side of the triceps, through which a probe could be passed to the bone. The operation was performed on January 12, in the ordinary manner. The wound healed kindly, and the patient is nearly ready to leave the hospital.

James Alexander, æt. nine, from Arbroath, entered the hospital on the 2d February, on account of a disease of the elbow-joint,

* I was informed to-day that, her father having lately died, leaving a widow and six children in very destitute circumstances, she is able to contribute towards their support by tambouring muslin.

under which he had laboured eighteen months. The bone can be felt extensively diseased; and the case seems in all respects a favorable one for excision, which will be performed so soon as the parents are informed and give their consent.

James Dennet came from Dundee to place himself under my care on account of pain, swelling, and redness over the olecranon, which had resulted from a blow on the elbow, received several months previously. On coming to town, he was persuaded to apply to another practitioner, who made a long incision through the skin. When he at length applied to me, a small part of the wound remained open over the olecranon, where there was some swelling and much tenderness on pressure. The patient declared that he was not any better than when he left home. Finding that a probe could be passed to the bone and a little way into its substance, I concluded that a superficial caries of the olecranon was the cause of his distress, and therefore, after exposing the bone, removed the softened portion with a gouge, on the 9th January. The wound is now nearly healed, and the patient makes no complaint.*

---

### *Excision of the Knee-joint.*†

The knee-joint, so far as regards its structure, is an equally favorable subject for excision with the elbow, since there is only one articulation concerned in the disease or affected by the operation, and not a number, as is the case in the wrist or ankle. But the advantages from the operation in this situation are much more questionable than in the shoulder or elbow, since not only is there much less difference between the utility of a natural leg and a wooden one, than between that of a real and artificial arm, but doubts may even be entertained as to the probability of deriving any assistance in progressive motion from the limb, which is preserved by cutting out the knee-joint. With the exception of the two cases operated upon by Mr. Park, of Liverpool, nearly fifty years ago, and the two cases lately published by Mr. Crampton, of Dublin, I am not acquainted with any recorded facts to guide us in deciding this question. Each of these gentlemen lost one of their patients, but the others survived and retained limbs so useful that the owners would not readily have exchanged them for artificial ones. Mr. Park's patient, a sailor, was able to ascend the rigging of his ship with the agility peculiar to that profession; and the woman on whom Mr. Crampton operated could walk the distance of eight or nine miles without suffering fatigue or inconvenience.

* M. Roux has removed the elbow-joint four times. One case terminated fatally; in the others the patients were never in danger, and they regained a partial use of the limb. Vide London Med. and Phys. Journal, April 1830, p. 368.—Editor.

† Ibid.

The advantages attending excision of the knee-joint over amputation in the thigh, in addition to the satisfaction of saving a limb, and promoting the credit of surgery, seem to me, first, the negative one of saving the patient from the inconvenience of resting his weight upon the *face* of a stump: secondly, the positive one of of preserving for him the tarsus, metatarsus, and toes, which constitute an apparatus much more efficient in protecting against the effects of concussion than any artificial one that can be constructed. Influenced by these considerations, I resolved to try the operation in some of those cases of diseased knee which so frequently result from white swelling in young subjects, and are condemned without any ceremony to amputation.

John Arnot, æt. eight, was admitted on the 1st December. His left knee was very much enlarged, and immoveably bent at an acute angle with the thigh. There were two sinuses on the inner side of the joint, which allowed a probe to reach the bone. The disease resulted from a fall on the ice, and was of three years' duration. His health was broken, and he seemed devoted to speedy destruction, unless something was done for his relief.

On Monday, therefore, the 7th December, I made two incisions across the fore part of the joint, extending from one condyle of the femur to the other, meeting at their extremities, and including the patella between them. The integuments thus insulated being removed, together with the patella, which was very much diseased, I exposed the extremity of the femur, and sawed it off. In doing this, the periosteum was separated from the bone, to which it adhered very slightly for about an inch, or rather more, and I therefore thought it right to saw off another portion to this extent. The head of the tibia was next exposed, and removed by means of cutting-pliers. One of the articular arteries was tied, and we then proceeded to dress the wound; but here an unexpected difficulty occurred, owing to the hamstring muscles, which, as already stated, were much contracted, and still prevented the limb from being straightened, notwithstanding the relaxation they had suffered in consequence of the removal of the joint. I extended the limb as far as was practicable, and secured it in this position by a splint and bandage. The patient had very little constitutional disturbance, but the wound presented a dry and unpromising appearance. The tibia, from not resting in opposition to the femur, was drawn up behind that bone, distended the integuments, and threatened to exfoliate extensively. I at length succeeded, by cautious extension and counter-extension, in reducing the displaced extremities of the bones, when the limb became quite straight, and the tendency to dislocation almost entirely ceased. The cure afterwards advanced satisfactorily, notwithstanding the most vexatious opposition on the part of the patient, who was a boy of uncommon quickness, but most perverse disposition.

Four weeks after the operation, the wound was all but healed, and the limb is now becoming every day more useful to the patient, who can already make considerable use of it in walking, though not yet provided with a high-heeled shoe.

Ann Mackintosh, æt. seven, entered the hospital on the 14th December, on account of a white swelling of the right knee, which had existed eighteen months, and was now in its last stage. There was a large sinus above the inner condyle, through which I introduced my finger into the joint, and felt it extensively diseased. Encouraged by the success of the former case, I performed a similar operation in this one on the 28th December, and think it unnecessary to mention the particulars, as they were in all respects similar to those already detailed. The articulating portions of bone removed were, as in the boy, extensively ulcerated and carious; but the soft parts were much less swelled, and less altered by the gelatinous degeneration of scrofulous action, than in him; the result, therefore, was expected to be, if possible, still more satisfactory.

Great difficulty was experienced, from contraction of the hamstrings, in preventing dislocation of the bones, and the femur, so far as it was visible, presented a bare and deadlike surface, but the favorable termination of the first operation, notwithstanding appearances equally disagreeable, prevented me from abandoning my sanguine expectations of success in this instance also. On the 6th of January, in order to prevent displacement of the bones, which all our efforts hitherto had been unable to effect completely, I cut away about two inches of the femur with the cutting-pliers, and then observed, to my extreme concern, that the bone was denuded beyond the farthest extent to which my finger could reach. Amputation now seemed to afford the only chance; but, before having recourse to it, I resolved to wait a little, in the expectation of nature pointing out at what part of the limb the operation ought to be performed. On the morning of the 8th, I found her very weak: she sunk rapidly, and died at two the same day.

I do not think that the enemies of excision of the knee-joint can found any thing upon this case, since it would appear from reasoning, and has been in great measure proved by experience, that excision of a joint is less dangerous than amputation of the limb; and the only question that can be agitated in respect to the merits of the operation in this situation concerns the utility of the limb which is preserved.*

* M. Roux deprecates excision of the knee-joint. He performed this operation in one case; the patient died on the nineteenth day. London Med. and Phys. Journal, April 1830, p. 368.—Editor.

### *Stricture of the Rectum, with Fistula in Ano opening into the Rectum.**

Robina Wright, admitted January 7th, three years ago began to suffer from pain in going to stool, with frequent desire to do so, and copious slimy discharge. She was then a servant in town, and up to that time had enjoyed good health.

To obtain relief from the complaints just mentioned, she entered the Royal Infirmary, and, with the exception of one week, remained there ever since (three years,) as a physician's patient.

She complained of excruciating pain on going to stool, with an almost incessant call to do so. There was a copious discharge of slimy matter from the rectum, but its solid contents were never passed of a larger size than that of a quill. To relieve her distress, she had been in the habit of taking three or four grains of opium daily, with a proportional quantity of the same introduced into the rectum. I found, on examination, a stricture of the rectum three inches from the orifice, so tight that the point of my finger could not pass through it. There was also a wide fistulous canal leading from the rectum into the vagina, and opening just within the orifice of each. Taking into account the youth of the patient, and the absence of any induration or thickening of the coats of the gut, I readily consented to attempt her cure.

I began by removing the stricture, which yielded without any difficulty to the introduction of steel bougies every third or fourth day. I then laid the fistula open, and she is now nearly well, being altogether free from pain in going to stool, and having no particular frequency of desire to do so.

The amount of relief experienced in this case can hardly be conceived by any one who had not an opportunity of seeing the patient. The agonizing pain she constantly endured was plainly depicted in her countenance, and the fetid matter incessantly escaping through the fistula into the vagina, not only rendered her existence still more wretched, but made her an insufferable nuisance to others.

* Ibid.

